# Prediction of Nitrogen Responses of Corn by Soil Nitrogen Mineralization Indicators

**DOI:** 10.1100/tsw.2001.329

**Published:** 2001-11-09

**Authors:** R.R. Simard, N. Ziadi, M.C. Nolin, A.N. Cambouris

**Affiliations:** Soil Science Department, University of Manitoba, Winnipeg, Canada; Soils and Crops Research Centre, Agriculture and Agri-Food Canada, 2560 Hochelaga Blvd, Ste-Foy, Quebec, Canada G1V 2J3,

**Keywords:** soil N mineralization potential, N rate to reach maximum corn grain yield, corn, spatial variability, nitrogen use in agricultural fertilization, environmental indicators, precision agriculture

## Abstract

Soil nitrogen mineralization potential (N_min_) has to be spatially quantified to enable farmers to vary N fertilizer rates, optimize crop yields, and minimize N transfer from soils to the environment. The study objectives were to assess the spatial variability in soil N_min_ potential based on clay and organic matter (OM) contents and the impact of grouping soils using these criteria on corn grain (Zea mays L.) yield, N uptake response curves to N fertilizer, and soil residual N. Four indicators were used: OM content and three equations involving OM and clay content. The study was conducted on a 15-ha field near Montreal, Quebec, Canada. In the spring 2000, soil samples (n = 150) were collected on a 30- x 30-m grid and six rates of N fertilizer (0 to 250 kg N ha^-1^) were applied. Kriged maps of particle size showed areas of clay, clay loam, and fine sandy loam soils. The N_min_ indicators were spatially structured but soil nitrate (NO3^–^) was not. The N fertilizer rate to reach maximum grain yield (N_max_), as estimated by a quadratic model, varied among textural classes and N_min_ indicators, and ranged from 159 to 250 kg N ha^-1^. The proportion of variability (R^2^) and the standard error of the estimate (SE) varied among textural groups and N_min_ indicators. The R^2^ ranged from 0.53 to 0.91 and the SE from 0.13 to 1.62. Corn grain N uptake was significantly affected by N fertilizer and the pattern of response differed with soil texture. For the 50 kg N ha^-1^ rate, the apparent N_min_ potential (ANM) was significantly larger in the clay loam (122 kg ha^-1^) than in the fine sandy loam (80 kg ha^-1^) or clay (64 kg ha^-1^) soils. The fall soil residual N was not affected by N fertlizer inputs. Textural classes can be used to predict N_max_. The N_min_ indicators may also assist the variable rate N fertilizer inputs for corn production.

### INTRODUCTION

The prediction of the soil nitrogen mineralization potential ($N_{min}$) in space and time in cool and humid climates is unreliable[1]. The soil N contents as evaluated by chemical extraction (e.g., KCl) show short-range variation and often poor spatial dependence[2]. Good estimates of $N_{min}$ are necessary to minimize N transfer to the environment, to design better fertilizer N recommendations, and improve N management in the crop-soil system[3]. Studies conducted in Quebec on corn reported that fertilizer N recoveries vary between 40 to 65% of applied N[4,5]. The nitrate ($NO_3^-$) concentration in the tributaries of the St. Lawrence is increasing[6] and 41% of the farmland area is intermediate to high risk of water contamination by N[7]. It is therefore important to achieve a good prediction of $N_{min}$ to minimize the risk of N transfer in corn production.

Prediction of $N_{min}$ may be achieved by integrating fairly static soil attributes such as organic matter (OM) and clay contents[8]. These attributes are spatially dependent in the St. Lawrence lowlands[9,10]. The prediction of fertilizer N needs by $N_{min}$ indicators can be used with Precision Agriculture (PA) technologies to define optimal N management strategies for corn.

Small plot research indicated that soil $N_{min}$ in cool and humid climates may be influenced by previous crop, soil mineral N content in spring, tillage system, landform, and soil drainage[3]. However, OM and soil N content remain key parameters in predicting N fertilizer needs[8,11]. The organic N pool in arable soils tends to be related to their clay content[5]. Until recently, in central Canada, N fertilizer application for corn was uniform[12,13] despite high within-field variability in crop N uptake[10,14]. Site-specific fertilizer N may increase crop yields and reduce residual soil N and subsequent leaching[15]. The objectives of our study were to (1) evaluate the spatial variability of the potential of $N_{min}$ based on clay and OM contents and (2) evaluate the impact of grouping soils using these criteria on corn grain yield, grain N uptake response curves to N fertilizer, and soil residual N.

### MATERIAL AND METHODS

#### Site Description

A 15-ha plot (270 × 555 m), part of a 34-ha field located in St. Marc-sur-Richelieu, near Montreal, province of Quebec, Canada, was selected for this study. Mean annual precipitation is around 1000 mm and near 2700 corn heat units are available in the growing season. An intensive soil survey (scale 1:7500) was conducted in 1997. Seven soil mapping units (eight polygons) were delineated (Fig. 1) and five soil series formed on clay and alluvium deposits were identified: Saint-Urbain (very-fine clayey, mixed, mesic Typic Humaquept), Providence (very-fine clayey, mixed, mesic Aeric Humaquept), Du Jour (fine-clayey, mixed, mesic Aeric Humaquept), Du Contour (coarse-loamy over clayey, mixed, mesic Humic Endoaquept), and Latour (coarse-loamy over clayey, mixed, mesic Aquic Dystrochrept). Drainage conditions range from imperfect to very poor.

#### Crop Management Information

The studied field is under a corn-soybean rotation. Starter fertilizer was 15 kg N $ha^{-1}$ applied as 18-46-0 on May 17, 2000. The treatments consisted of six rates of N (0, 50, 100, 150, 200, and 250 kg N $ha^{-1}$) applied as anhydrous ammonia on July 5, 2000. The treatments were distributed in a randomized complete block design with five replicates for 30 plots of 9-m wide × 555-m long. Corn was harvested in November $7^{th}$, 2000 at each sampling point from a 5- × 0.75-m area to determine entire above ground biomass. Only corn grain yield adjusted to 14% moisture content was presented in this study.

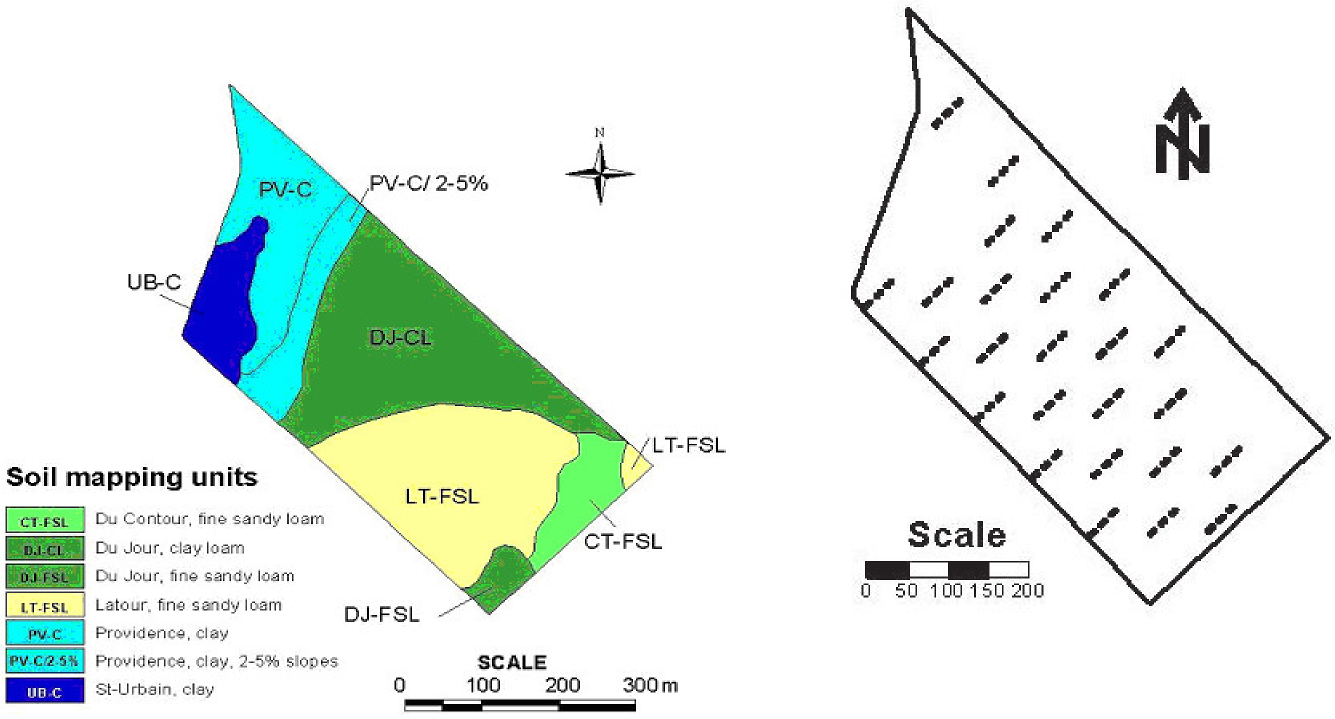

A B

**FIGURE 1**. Maps showing (A) soil mapping units and (B) sampling design (n = 150) of the experimental plot.

### Soil Sampling Strategy

In April 2000, a stratified systematic soil sampling (n = 150) was superimposed on the experimental design. Each block of six strips was positioned to cross the soil mapping units. In each strip, five subsamples were systematically taken on a 100-m interval using a triangular grid design between blocks to obtain 150 sampling grid points (30 plots * 5 subsamples). At each sampling point (n = 150), four cores (one in each quadrant of a 1.5-m radius circle), 0.2-m deep, were compounded to obtain a representative sample. Additional sampling points (50) located within and outside the experimental plot were used to improve the interpolation by kriging.

### Soil Analysis

Samples were air dried, ground to <2 mm, sieved, and analyzed for the selected chemical and physical properties. Soil pH was measured in distilled water with a 1:2 soil:solution ratio. OM content was determined by wet oxidation[16]. Particle size analysis was carried out by the hydrometer method after organic matter oxidation[17]. The soil $NO_3$-N and $NH_4$-N contents were extracted in 2 *M* KCl with a 1:10 soil:solution ratio[18]. The $NO_3$-N in the extracts was measured using a Dionex 4000i liquid chromatograph equipped with Ion Pack CG5 and CS5 columns (Dionex Corp., Sunnyvale, CA), whereas $NH_4$-N was determined colorimetrically by the indophenol blue method[18].

### Data Analysis

Descriptive statistics and regression analysis were calculated using SAS[19]. Geostatistical analysis (semivariance and block kriging) were performed using GS+[20] and kriged maps were produced with Surfer[21]. Analysis of variance on the effects of N fertilizer on corn grain yield and N uptake and on soil residual N was performed using the GLM procedure of SAS[19]. The N fertilizer rate ($N_{max}$) required to reach the maximum corn grain yield was estimated by a quadratic model and four $N_{min}$ indicators as follows:

$$\Delta Y = a + bX + cX^2$$

where DY is corn grain yield (yield at a given N rate applied – yield with no N applied) in Mg $ha^{-1}$, X the N fertilizer rate (kg $ha^{-1}$), and a, b, and c are regression constants. The $N_{max}$ was estimated by setting the first partial derivative of the N response curve equal to zero:

$$d\Delta Y/dX = b + 2cX = 0$$

and solving for X. Therefore, $N_{max}$ (kg N $ha^{-1}$) is calculated as:

$$N_{max} = -b/2c$$

The $N_{max}$ was not estimated when c was positive. When $N_{max}$ was >250 kg $ha^{-1}$, 250 kg $ha^{-1}$ were used[22]. The coefficient of determination ($R^2$) and the standard error of the estimate (SE) were used to determine $N_{max}$ in each soil class using OM and three equations involving OM (g $kg^{-1}$) and clay content (g $kg^{-1}$) as $N_{min}$ indicators. Three soil OM classes were thus obtained[23]: OM ≤ 25, 25 < OM < 35, and OM ≥ 35. The three following equations[10] were used to arbitrarily define three classes as follows:

$N_{min1}$ (kg $ha^{-1}$) = 4.00 + 0.29 OM + 0.22 clay;
$N_{min1} \le 50$, $50 < N_{min1} < 100$, and $N_{min1} \ge 100$.

$N_{min2}$ (kg $ha^{-1}$) = 22.16 + 1.91 OM;
$N_{min2} \le 70$, $70 < N_{min2} < 90$, and $N_{min2} \ge 90$.

$N_{min3}$ (kg $ha^{-1}$) = 18.62 + 1.46 OM + 0.10 clay;
$N_{min3} \le 70$, $70 < N_{min3} < 100$, and $N_{min3} \ge 100$.

An apparent $N_{min}$ coefficient (ANM) was also calculated as follows: ANM (kg $ha^{-1}$) = (Corn grain N uptake + [Fall soil N-Spring soil N] – Fertilizer N added); N uptake was calculated by multiplying corn grain yield by its N concentration.

An apparent N recovery coefficient ($N_{rec}$) was calculated as: $N_{rec}$ (%) = (Corn grain N uptake from a N fertilizer treatment – N uptake from the control)* 100 /Fertlizer N added.

## RESULTS AND DISCUSSION

### Within-Field Spatial Variability

Within-field variability of soil attributes (Table 1) is larger than reported for other intensively sampled fields from the St-Lawrence Lowlands[9,10]. This may be related to different soil types developed from contrasting parent materials (marine clay and loamy alluvium) and drainage classes. Kriged maps of particle size showed three soil classes: clay, clay loam, and fine sandy loam soils. Clay content ranges from 104 to 586 g $kg^{-1}$ (CV = 52%) and sand from 27 to 724 g $kg^{-1}$ (CV = 45%). The OM content is moderately variable (CV = 22%) ranging from 16 to 57 g $kg^{-1}$. $N_{min1}$ is more variable (CV = 45%) than $N_{min2}$ (CV = 16%) or $N_{min3}$ (CV = 22%). The $N_{min3}$ estimates a higher mean soil N mineralization than $N_{min2}$ and $N_{min1}$.

Isotropic semivariograms were computed to assess the within-field spatial continuity in $N_{min}$ indicators: clay, OM, $NH_4^+$, $NO_3^-$, and $N_{min1}$ to $N_{min3}$. Spherical models were most often selected (Table 2). The semivariograms show very good fits ($R^2$ > 0.90) with isotropic models and relatively low residual sums of squares (data not shown). All variables except for $NO_3^-$ show high $C/C_0 + C$ ratios (>0.75) indicating that the structured variance (C) is a high proportion of the total variation (sill).

This suggests that the sampling strategy precisely and reliably modeled the spatial variability. However, the short-range variability in $NO_3^-$ content indicates that a finer grid size is necessary to model its spatial distribution. This is confirmed by the cross-validation analysis (CVA) (Table 2). The indicators of soil $N_{min}$ based on clay content (clay, $N_{min1}$, and $N_{min3}$) show higher $R^2$ from the CVA than those derived from OM (OM and $N_{min2}$). The $NH_4^+$ has a higher $R^2$ than $NO_3^-$ which is particularly very low (0.20). The range ($A_0$), the maximum distance over which properties remain spatially correlated, is quite small (42 to 100 m) for all indicators, which suggest that a more intensive soil sampling than the 100- × 100-m grid common to commercial survey programs is needed to reliably estimate these $N_{min}$ indicators in this field. The soil $NO_3^-$ content, commonly used for N fertilizer pre-

**TABLE 1**
**Statistical Description of Some Physico-Chemical Properties of the 0- to 20-cm Soil Layer of the Experimental Field at the Beginning of the Study (n = 150)**

| Variable | Mean | Range | CV (%) |
|---|---|---|---|
| Sand (g $kg^{-1}$) | 467 | 27–724 | 45 |
| Silt (g $kg^{-1}$) | 259 | 163–414 | 27 |
| Clay (g $kg^{-1}$) | 274 | 104–586 | 52 |
| OM (g $kg^{-1}$) | 29 | 16–57 | 22 |
| $NO_3^-$ spring (kg $ha^{-1}$) | 83 | 13–210 | 16 |
| $NO_3^-$ fall (kg $ha^{-1}$) | 164 | 64–304 | 23 |
| $N_{min1}$ (kg $ha^{-1}$) | 74 | 35–145 | 45 |
| $N_{min2}$ (kg $ha^{-1}$) | 77 | 52–131 | 16 |
| $N_{min3}$ (kg $ha^{-1}$) | 88 | 63–140 | 22 |

**TABLE 2**
**Geostatistical Parameters**

| Variable | Model | Ratio* $C/C_0 + C$ | Range $A_0$ (m) | Model fits ($R^2$) | Cross-Validation ($R^2$) |
|---|---|---|---|---|---|
| Clay | Spherical | 1.00 | 57 | 0.94 | 0.95 |
| OM | Spherical | 0.88 | 72 | 0.95 | 0.66 |
| $NH_4^+$ | Spherical | 0.76 | 80 | 0.99 | 0.81 |
| $NO_3^-$ | Exponential | 0.50 | 44** | 0.92 | 0.20 |
| $N_{min1}$ | Exponential | 1.00 | 42** | 0.94 | 0.96 |
| $N_{min2}$ | Spherical | 0.87 | 72 | 0.95 | 0.66 |
| $N_{min3}$ | Spherical | 1.00 | 100 | 0.92 | 0.93 |

*Note*: n = 150; lag class interval = 10 m; active lag distance = 90 m; number of pairs per variogram class interval = 40 to 361.

* Proportion of the sill ($C_0 + C$) explained by spatially structured variance (C) where $C_0$ is the nugget variance.

** For exponential model, the effective range is defined as 3 $A_0$, the distance at which the sill is within 5% of the asymptote.

scription, is inadequate here to guide variable N fertilizer application of since it did not show a defined spatial structure.

### Corn Grain and N Uptake Response to N Fertilizer

N fertilizer significantly ($p < 0.001$) increased corn grain yield (Table 3). Averaged across all soil types, grain yield was 4.00 Mg $ha^{-1}$ in control areas (0 N) and 6.65 Mg $ha^{-1}$ where 250 kg N $ha^{-1}$ was applied. A moderate corn grain yield variability (CV = 26.7%) was observed with a minimum of 1.4 Mg $ha^{-1}$ and a maximum of 8.7 Mg $ha^{-1}$. The highest average yield (6.48 Mg $ha^{-1}$) was observed on the fine sandy loam and the lowest one on the clay (4.86 Mg $ha^{-1}$). The corn grain N uptake was also significantly affected by N fertilizer and the pattern of response differed between soil types. The maximum N uptake (83 kg N $ha^{-1}$) was obtained with 250 kg N $ha^{-1}$ on the clay but with only 150 kg N $ha^{-1}$ on the clay loam and fine sandy loam. For the 50 kg N $ha^{-1}$ rate, the ANM (as estimated by the N uptake from the control treatment and the difference between fall and spring N content in the 0- to 0.2-m layer) was significantly larger in the clay loam (122 kg $ha^{-1}$) than in the fine sandy loam (80 kg $ha^{-1}$) or clay (64 kg $ha^{-1}$). This suggests that the contribution of clay content to ANM as suggested from incubation studies[10] did not hold under the climatic conditions of 2000. These ANM are however in the same range as the $N_{min}$. The clay area has a larger average OM content (34.2 g $kg^{-1}$) than the loamy soils (27.2 g $kg^{-1}$). It should theoretically have a higher ANM than coarser soils. These low ANM for the clay area are probably related to the wet soil conditions that may have restricted N mineralization[3] or enhanced N losses due to leaching and/or denitrification. Braum et al.[24] indicated that the relationship between N fertilizer response and soil attributes was weather-dependent.

### The $N_{max}$ as Predicted by N Mineralization Indicators

The estimated $N_{max}$ varied with $N_{min}$ indicators and ranged between 159 and 250 kg ha$^{-1}$ (Table 4). The highest $N_{max}$ was observed in the clay area that had also the lowest ANM (Table 3). The year 2000 climatic conditions may partly explain this variation. Sogbedji et al.[25] reported that economic optima N fertilizer for corn are strongly affected by weather especially by early season precipitation. The April-May rainfall was much larger in 2000 (249 mm) than the last 30-year average (153 mm). Precipitation pattern for the remainder of the growing season was, however, comparable to the average of the last 30 years. Denitrification and N leaching transfers increase under these very wet spring conditions[26] and therefore may have resulted in larger $N_{max}$ on clay than in better drained coarser soils. Waterlogging is also known to limit $N_{min}$ from the soil organic N pool and nitrification[3]. These results also indicate that the locally recommended 170 kg N ha$^{-1}$ application would have been adequate to reach maximum yield on the loamy soils but not on the clay. The $N_{max}$ calculated over the whole field was very close to the recommended rate for corn[12].

The $R^2$ and SE of the quadratic model used to determine $N_{max}$ varied among textural soil groups and $N_{min}$ indicators

**TABLE 3**
**Corn Grain Yield, N Uptake, ANM, and $N_{rec}$ as Estimated for the Three Soil Classes (C = Clay; CL = Clay Loam; FSL = Fine Sandy Loam)**

| N Rates | Corn Grain Yield (Mg ha$^{-1}$) | | | Corn N Uptake (kg ha$^{-1}$) | | | ANM (kg ha$^{-1}$) | | | $N_{rec,\%}$ | | |
|---|---|---|---|---|---|---|---|---|---|---|---|---|
| (kg N ha$^{-1}$) | C | CL | FSL | C | CL | FSL | C | CL | FSL | C | CL | FSL |
| 0 | 3.65 | 3.72 | 4.86 | 42 | 36 | 50 | 93 | 140 | 135 | – | – | – |
| 50 | 4.31 | 6.09 | 6.36 | 46 | 66 | 70 | 64 | 122 | 80 | 8.0 | 60.0 | 40.0 |
| 100 | 4.98 | 6.09 | 6.82 | 57 | 64 | 78 | 33 | 43 | 57 | 15.0 | 28.0 | 28.0 |
| 150 | 5.42 | 6.70 | 6.89 | 55 | 82 | 86 | 5 | –8 | 18 | 8.7 | 30.7 | 24.0 |
| 200 | 4.40 | 6.36 | 6.83 | 51 | 71 | 81 | –61 | –79 | 2 | 4.5 | 17.5 | 15.5 |
| 250 | 6.42 | 6.42 | 7.10 | 83 | 75 | 85 | –69 | –59 | –90 | 16.4 | 15.6 | 14.0 |
| Means | 4.86 | 5.90 | 6.48 | 56 | 66 | 75 | 11 | 27 | 34 | 10.5 | 30.4 | 24.3 |

Mean square value ($\times 10^{-3}$)

| Source of variation | | | | |
|---|---|---|---|---|
| Replicates | 2.19 | 0.74* | 23.17*** | 3.03** |
| N rates | 19.49*** | 4.33*** | 145.53*** | 2.27** |
| Soil class | 26.74*** | 2.83*** | 10.25** | 3.78** |
| Error | 0.63 | 322.09 | 2538.18 | 725.36 |

*, **, ***: significant at $p < 0.05$, $p < 0.01$, $p < 0.001$, respectively.

**TABLE 4**
**Fertilizer N Rate to Reach $N_{max}$, $R^2$, and SE as Calculated by Quadratic Model using Four N Mineralization Indicators**

| | Soil Classes* | | | OM (g kg$^{-1}$)** | | | $N_{min1}$ (kg ha$^{-1}$)*** | | | $N_{min2}$ (kg ha$^{-1}$)† | | | $N_{min3}$ (kg ha$^{-1}$)† | | |
|---|---|---|---|---|---|---|---|---|---|---|---|---|---|---|---|
| | $N_{max}$ | $R^2$ | SE | $N_{max}$ | $R^2$ | SE | $N_{max}$ | $R^2$ | SE | $N_{max}$ | $R^2$ | SE | $N_{max}$ | $R^2$ | SE |
| **C** | 250 | 0.64 | 0.664 | 227 | 0.60 | 0.594 | 187 | 0.89 | 0.090 | 226 | 0.61 | 0.594 | 166 | 0.68 | 0.488 |
| **CL** | 192 | 0.87 | 0.311 | 176 | 0.88 | 1.392 | 159 | 0.85 | 0.411 | 171 | 0.87 | 0.357 | 185 | 0.90 | 0.243 |
| **FSL** | 173 | 0.91 | 0.130 | 250 | 0.53 | 1.626 | 250 | 0.58 | 0.859 | 250 | 0.53 | 1.390 | 250 | 0.55 | 0.879 |
| **Global** | 180 | 0.87 | 0.31 | 180 | 0.87 | 0.310 | 180 | 0.87 | 0.310 | 180 | 0.87 | 0.310 | 180 | 0.87 | 0.310 |

* C = clay; CL – clay loam; FSL – fine sandy loam.
** 1 = OM < 25; 2 = 25 < OM < 35; 3 = OM > 35.
*** 1 = <50; 2 = 50 to 100; 3 = >100.
† 1 = <70; 2 = 70 to 90; 3 = >90

Global: all data; $N_{min1}$ = 4.00 + 0.29 OM + 0.22 clay; $N_{min2}$ = 22.16 + 0.91 OM; $N_{min3}$ = 18.62 + 1.46 OM + 0.10 clay; $N_{min1}$, $N_{min2}$, and $N_{min3}$ are calculated according to Simard and N'dayegamiye[10].

(Table 4). The $R^2$ ranged from 0.53 to 0.91 and the SE from 0.13 to 1.62. The highest $R^2$ and the lowest SE were obtained on the fine sandy loam area. Based on these two parameters, $N_{max}$ was 250 kg N ha$^{-1}$ on clay, 192 kg N ha$^{-1}$ on clay loam, and 173 kg N ha$^{-1}$on the fine sandy loam. The $N_{min1}$ was the best $N_{min}$ indicator to determine $N_{max}$ with on average a $R^2$ of 0.77 and a SE of 0.45.

### Fertilizer N Recovery and Residual Soil N

The N fertilizer recovery coefficient in grain ($N_{rec}$) was affected by soil texture and N fertilizer rates. Averaged across N rates, it was 10.5% in the clay, 30.4% in the clay loam, and 24.3% in the fine sandy loam area (Table 3). These values are lower than in previous studies[4,5]. The $N_{rec}$ was inversely proportional to fertilizer N rates on the clay loam and fine sandy loam areas but not related to it in the clay area. Wet soil conditions may explain the low recovery of ANM on the clay soil that resulted in a higher $N_{max}$ than in the other two areas. The residual soil N was not significantly affected by the N fertilizer treatments and the average residual N in the 0- to 0.2-m layer was 81 mg kg$^{-1}$ (data not shown). The absence of difference in fall soil residual N between fertilizer rates confirm that care should be taken not to overfertilize these soils since any excessive N may be transferred from the soil profile to the adjacent environment as it was not found as fall residual soil N.

### CONCLUSIONS

The prediction of $N_{min}$ involves the measurement of biological and physical processes. This is a very difficult task. Indicators of $N_{min}$ based on soil OM and clay contents showed a spatial structure in the studied field but soil $NO_3^-$ content did not. The corn grain yield, grain N uptake, fertilizer response, and N recovery were significantly different between areas of contrasting soil texture. Conversely, the fall residual N was not affected by N inputs or textural class. The $N_{max}$ was larger on the clay than in other soils presumably because of low apparent $N_{min}$ and fertilizer $N_{rec}$. Although the results of the present study may be specific to the soil and climatic conditions, they suggest that management units based on soil texture class would help the management of fertilizer N in corn production on these poorly drained soils. This would increase economical benefits of N application and reduce the potential transfer of N to the atmosphere and to surface and ground waters.

### ACKNOWLEDGMENTS

This study is funded by Nutrite Inc., La Ferme Promarc, Innotag Inc., and Agriculture and Agri-Food Canada through a matching investment initiative program. The assistance of Guy Forand, Christian Beaudry, Alain Larouche, Mario Deschenes, Nicolas Samson, and Lucie Grenon is greatly appreciated.

| | | |
|---|---|---|
| Received: | July | 19, 2001 |
| Revised: | October | 10, 2001 |
| Accepted: | October | 26, 2001 |
| Published: | November | 9, 2001 |
